# Heavy Metals in Agriculture: Sources, Industrial Applications, Plant Toxicity, and Remediation Approaches

**DOI:** 10.3390/ijms27146192

**Published:** 2026-07-10

**Authors:** Muhammad Musa Khan, Baoli Qiu, Zengrong Zhu

**Affiliations:** 1 Seed Engineering and Plant Quarantine, Hainan Institute of Zhejiang University, Yazhou Bay Science and Technology City, Sanya 572000, China; 2 Engineering Research Centre of Biotechnology for Active Substances, Ministry of Education, Chongqing Normal University, Chongqing 401331, China; baoliqiu@cqnu.edu.cn

**Keywords:** heavy metals, abiotic stress, phytoremediation, agro-ecosystem, environmental toxicity

## Abstract

Heavy metal pollution has become a critical concern in agricultural ecosystems driven by a complex matrix of industrial practices, high-input fertilizers, metal-based agrochemicals, and wastewater irrigation. While the previous literature typically highlights general physiological symptoms of heavy metal stress, this review provides a novel, comprehensive framework that bridges three independent pillars: specific industrial applications dictating elemental pathway, localizes active root-zone transport kinetics, and an engineering-based evaluation of emerging remediation strategies. We systematically synthesized literature from 2000 to 2026 across major databases (WoS, PubMed and Google Scholar), applying strict inclusion criteria based on data validation, experimental reproducibility, and mechanistic depth. We examine the geochemical behavior, cellular toxicity, and plant resilience mechanics of seven priority elements like cadmium, lead, arsenic, aluminum, mercury, chromium and molybdenum. Rather than merely reiterating superficial visual damage like chlorosis or stunted growth, we focus on physiological and molecular root causes of phytotoxicity, including the structural hijacking of essential nutrient networks, intracellular reduction cascades and organelle-specific oxidative disruption. This review also discussed the discovery of specialized, energy-dependent eukaryotic transport mechanisms like ABC transporters and a comparative operational blueprint evaluating physical–chemical conventional remediation techniques against advanced in situ and ex situ biotechnological approaches, including biochar assistance, microbial engineering, rhizosphere synergies, and engineered nanomaterials. By systematically linking industrial source dynamics with cellular toxicological mechanisms and field-scale engineering feasibility, this review establishes an actionable roadmap for future genetic, agronomic, and management interventions aimed at securing global food.

## 1. Introduction

The environment is becoming increasingly polluted due to substantial economic expansion and rapid development across sectors, including agriculture and industry [1]. Environmental pollutants are potentially deleterious substances that can infiltrate the ecosystem from natural and anthropogenic sources. Specific ecological activities, including synthetic industry production, coal conversion, and waste incineration, can pose significant threats to abiotic components (water, air, and soil) and biotic communities [2]. Heavy metals and pesticides are two examples of environmental pollutants that endanger ecosystems by seriously altering their structure and function [3]. Heavy metals are elements with high atomic weights and densities above 5 g/cm^3^; they are found in nature [4]. In contrast to their physical characteristics, heavy metals’ chemical properties are of paramount practical significance. Global think tanks are increasingly focused on environmental levels of toxicity that exceed set maximum residual limits (MRLs). Copper (Cu), Cadmium (Cd), zinc (Zn), and lead (Pb) are elements that contribute to significant environmental and health concerns [5,6]. The rapid rise in heavy metal pollution is causing significant damage, particularly to the agricultural economy. These toxins accumulate in soil, are taken up by plants, and thus enter the food chain [7]. As heavy metal pollution has reached emergency levels, implementing robust and practical remedies is essential to reduce the associated risks.

Heavy metals, while essential for the functioning of many organs in plants and humans, can be detrimental if their concentrations exceed permitted levels. A multitude of research investigations in this domain reveal that heavy metals can emanate from diverse sources, including mining, agriculture, and industry. Agricultural sources can be categorized into four groups: fertilizers, pesticides, livestock dung, and wastewater [7,8,9,10,11]. A survey indicated that agriculture and industry substantially contribute to heavy metal contamination in agricultural soil and plants, especially in soils adjacent to cement and electroplating companies [12]. The uppermost layer of soil is an optimal site for the accumulation of heavy metals, which are subsequently translocated to plant roots, where they are absorbed by water and disseminated throughout the plant via the vascular system.

The buildup of heavy metals in an environment might be regarded as an aggregation of components. The roots of plants serve as the contact site for the transport of heavy metal ions from the soil. They tend to bind and immobilize soil contaminants, thereby decreasing the bioavailability of pollutants. To mitigate heavy metal contamination, plants adopt different phytoremediation strategies. These exploit the natural physiological uptake and binding capabilities of plants to deal with heavy metal stress. These strategies include: (i) Phytoextraction: A technique in which plants absorb and translocate heavy metals from contaminated soil, concentrating them into their above-ground harvestable ground biomass. (ii) Phytostabilization: A strategy that leverages plants to immobilize heavy metals in soil, thereby reducing their mobility, bioavailability, and environmental migration risk. (iii) Rhizofiltration: A form of phytoremediation in which plant root systems absorb, sequester, or precipitate toxic substances, particularly heavy metals, from contaminated water or soil. Ultimately, when heavy metals accumulate beyond threshold limits, severe phytotoxicity occurs, which results in impairing the agro-ecosystem and adversely affecting human health [13,14]. 

**Figure 1:**
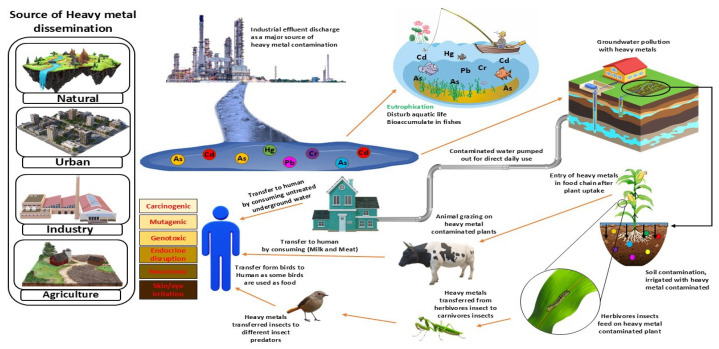
Anthropogenic sources, ecological pathways, and trophic transfer of heavy metal contamination in agricultural ecosystems. Industrial activities, urban mining operations, and municipal waste discharge toxic heavy metals directly into the atmosphere, soil, and aquatic repositories via factory smoke stacks and industrial effluent runoff. High-input agrochemical applications, including specialized chemical fertilizers, metal-based pesticides, sewage biosolids, and contaminated wastewater irrigation, enrich agricultural topsoils with highly bioavailable metallic fractions. These toxic elements are subsequently absorbed by crop root systems via localized rhizo-uptake, leading to intracellular accumulation that induces severe phytotoxicity characterized by leaf chlorosis and root growth inhibition. The bioaccumulation of toxic elements within food crops drives dangerous trophic transfer and biomagnification cascades across secondary ecological pathways, moving through phytophagous insects and domestic livestock to facilitate dietary exposure, food chain contamination, and severe health risks to human consumers. As, Arsenic; Cd, Cadmium; Hg, Mercury; Cr, Chromium; Pb, Lead.

Although a number of studies document the general symptoms of heavy metal stress or discuss cleanup methods in isolation, current reviews rarely connect the dots between industrial source chemistry, sub-cellular plant kinetics, and field-scale engineering reality; to bridge this gap, our review offers a unified, multi-tiered synthesis of seven priority toxic elements: cadmium, lead, arsenic, aluminum, mercury, chromium and molybdenum. We map out their industrial origins alongside the precise pathways they use to hijack essential plant nutrient transporters. From there, we provide a clear comparative framework that weighs traditional physical–chemical methods against advanced, targeted in situ biotechnological approaches. By systematically tracking the workflow from geochemical source to cellular mechanic and practical field execution, this review delivers a practical, cross-disciplinary roadmap for protecting global food safety and agricultural resilience (Figure 1).

## 2. Types of Heavy Metals in Agriculture and Their Industrial Usage

### 2.1. Arsenic

Arsenic is a ubiquitous trace element in the environment. Its most toxicologically important inorganic species are arsenite (As III) and arsenate (As V). Organic arsenic primarily exists as methylated metabolites: monomethylarsonic acid (MMA), dimethylarsinic acid (DMA), and trimethylarsine oxide. The contamination of the environment with arsenic may be traced to both natural processes and human activity [15]. Historically, several arsenic-containing compounds have been manufactured in industrial settings and heavily integrated into agricultural and medical goods, including insecticides, herbicides, fungicides, wood preservatives, algicides, dyestuffs, and sheep dips. They have also been utilized in veterinary medicine to eliminate tapeworms in sheep and cattle as well as in human medicine for treating syphilis, yaws, amoebic dysentery, and trypanosomiasis [16]. While most of these inorganic and medicinal applications have been globally restricted or phased out due to severe toxicity, arsenic contamination remains a concern for modern agriculture. This ongoing risk is driven by the highly persistent ‘legacy repositories’ of these historical applications bound to agricultural soils, alongside the continuing, legally regulated use of specific organic arsenicals like monosodium methanearsonate (MSMA) herbicides in cotton farming and turf management, and chromated copper arsenate (CCA)in industrial wood preservation [17]. Arsenic-based drugs are still employed in veterinary practice to treat parasitic infections like canine filariasis and blackhead disease in poultry [16]. In the treatment of certain tropical diseases, such as African sleeping sickness and amoebic dysentery, arsenic-based drugs are also still used. Arsenic trioxide was given the green light by the Food and Drug Administration (FDA) not too long ago to be used as an anticancer drug in treating acute promyelocytic leukemia [18]. It has been hypothesized that the therapeutic activity of this compound is due to the stimulation of programmed cell death (apoptosis) in leukemia cells [19]. 

### 2.2. Cadmium

As a persistent environmental toxin, cadmium threatens ecological integrity and public health. According to the ATSDR list, it is the sixth-most dangerous heavy metal. Cadmium occurs ubiquitously in the Earth’s crust at an average concentration of 0.1 mg/kg. Environmentally, its compounds are most concentrated in sedimentary rocks, with marine phosphates containing notably high levels of about 15 mg/kg [20]. Cadmium is widely used in a variety of industrial processes. Manufacturing alloys, pigments, and batteries are among cadmium’s most critical industrial applications. It is a by-product of the zinc manufacturing process, and people or animals can be exposed to it in the workplace or the natural environment. The daily cadmium consumption in the United States is around 0.4 micrograms per kilogram daily, less than half of the oral reference dosage established by the USEPA [21]. This decline is due to strict controls on industrial wastewater from plating companies and, more recently, to widespread bans on cadmium use in several countries. According to recent studies, the total area in China that is contaminated by cadmium is more than 11,000 hectares. The exposure to cadmium in the environment is somewhat higher in Japan and China than in any other country [22]. Because of the rapid pace at which it is transferred from the soil to the plants, cadmium is most commonly detected in fruits and vegetables [23]. Cadmium is a highly toxic, non-essential heavy metal that adversely affects plants by disrupting enzymatic processes, inducing oxidative stress, and causing nutritional deficiencies [24]. At the molecular level, cadmium (Cd^2+^) toxicity is driven by its ability to compete with and hijack essential nutrient network. Lacking dedicated uptake system, Cd^2+^ enters root cells opportunistically across the plasma membrane via low-specificity micronutrient transporters, primarily IRT1 (Iron-regulated transporter 1), NRAMP (Natural Resistance-Associated Macrophage Protein) family members and ZIP channels. Once inside the cytosol, plants coordinate homeostatic chelation by rapidly synthesizing sulfur-rich ligands like phytochelatins (PCs) and metallothioneins (MTs), which bind with free Cd^2+^ and are actively pumped into the vacuole via HMA3 (Heavy Metal ATPase 3) and ABCC transporters. When these sequestration limits are exceeded, free Cd^2+^ displaces essential metallic cofactors like Zn^2+^ and Fe^2+^ from metalloenzymes which causes the denaturation of proteins, depleting cellular glutathione pools, and generating ROS stress that activates the MAPK cascades downstream to initiate cell death [25]. 

### 2.3. Chromium

Naturally occurring quantities of the element chromium (Cr) are found in the crust of the Earth. The oxidation levels for this element, known alternatively as valence states, can range from Cr(II) all the way up to Cr(VI) [26]. The trivalent chromium Cr(III), is the most stable of the chromium compounds. It is also the form that naturally occurs in ores, such as ferrochromite. The state designated as hexavalent chromium Cr(VI) is the second-most stable of all possible configurations [27]. Natural environments do not produce chromium in its elemental form Cr (0). Many natural and anthropogenic sources release chromium into the atmosphere; however, industrial enterprises are primarily responsible for chromium discharge. Chromium may be found in the soil, water, and air. The most substantial contributors to chromium emissions include the manufacturing sectors for ferrochrome and chrome pigment, alongside metal processing, tannery facilities, chromate production, and stainless-steel welding. It has been determined that the release of chromium into the air and wastewater, primarily by the metallurgical, refractory, and chemical sectors, is to blame for the rise in ambient concentrations of chromium. Most chromium discharged into the environment due to human activities is hexavalent chromium Cr(VI) [28]. Regarded as a potent human carcinogen by multiple health authorities, Cr(VI) represents a significant and harmful environmental pollutant [29]. The risk to one’s health posed by exposure to chromium is variable depending on the element’s oxidation state. The metal form of chromium is relatively nonhazardous, but the hexavalent form is quite hazardous. Conventional scientific understanding held that Cr(VI) was exclusively anthropogenic, in contrast to the naturally occurring Cr(III) found throughout the environment. This view has been challenged by the discovery of geogenic Cr(VI) in aquifers and surface waters at levels above the WHO drinking water guideline of 50 µg/L [30]. Chromium is widely used in various industrial processes; therefore, it is a pollutant that can be found in many different environmental systems [31]. Chromium compounds have widespread applications in industry, including plating with chrome, leather tanning, producing dyes and pigments, and preserving wood. As an anticorrosive agent, chromium is utilized in cooking systems and boilers [32].

### 2.4. Lead

Naturally present in the earth’s crust in minute quantities, lead is a metallic element distinguished by its bluish-gray color. Despite its natural occurrence, emissions of lead are dramatically amplified by human actions such as the combustion of fossil fuels, mining operations, and various industrial production activities. Recognized as an extremely dangerous substance, the extensive historical and contemporary application of lead has led to severe pollution and adverse health outcomes across numerous global regions [33]. Lead is utilized in multiple applications throughout the industrial, agricultural, and residential sectors. Current industrial uses for this metal include the manufacture of lead-acid batteries, ammunition, various metallic items like solder and pipes, and protective devices that attenuate X-ray radiation. U.S. recent domestic consumption of refined lead has stabilized at approximately 1500 thousand metric tons annually. With this current supply chain, the production of lead-acid batteries represents the dominant sector, accounting for an estimated 67% of total domestic consumption. Due to recent advancements in the automotive sector, lead consumption has increased. In 2025, an estimated 1 million tons of secondary lead were produced, an amount equivalent to 70% of apparent domestic consumption. Nearly all secondary lead was recovered from old scrap, mostly lead-acid batteries [20,34]. Paints, ceramics, caulk, and pipe solders are just a few examples of industrial products that have seen a dramatic decrease in lead usage in recent years [35]. In spite of this advancement, it was revealed that out of the 16.4 million households in the United States that had more than one child younger than six years old in each household, twenty-five percent of those homes still contained large amounts of lead-contaminated paint, dust, or surrounding bare soil [36]. 

### 2.5. Mercury

Metallic mercury is a naturally occurring metal that is a silvery-white liquid that is odorless and glossy. When heated, it transforms into a gas that is odorless and colorless. Mercury has a high level of toxicity and highly bioaccumulative properties [37]. Classified as a dense transition metal on the periodic table, mercury possesses the unique characteristic of existing in three natural states: elemental, inorganic, and organic. Each of these forms has a distinct and specific toxicity profile [38]. Elemental mercury is a liquid at room temperature with a notably high vapor pressure, which facilitates its release into the atmosphere in the form of mercury vapor. In its ionic form, mercury exists with two possible oxidation states: a +1 (mercurous) state and a +2 (mercuric) state [39]. In the environment, the most widespread organic chemical form is methylmercury. This compound results from a biological methylation process, whereby microorganisms present in water and soil convert inorganic mercury [40]. Mercury is a prevalent environmental contaminant and toxicant that produces profound changes in the body’s physical tissues and is responsible for a wide variety of adverse health effects [41]. Mercury can be found in the environment in a variety of chemical forms, and both humans and animals are susceptible to its effects. Mercury exists in this category in multiple forms: as elemental vapor (Hg^0^), in the inorganic states of mercurous (Hg^+1^) and mercuric (Hg^+2^), and within various organic mercury compounds [42]. The presence of mercury in the environment is so pervasive that it is impossible for any living thing, including humans, plants, and animals, to avoid being exposed to mercury in any form [43].

Mercury serves functional roles in a wide array of industrial contexts. This includes its use in electrical components such as switches, thermostats, and batteries, its application in dental amalgams for restorative work, and its involvement in diverse processes like caustic soda production in nuclear reactors, antifungal treatment of wood, solvent extraction for reactive and precious metals, and preservation in the pharmaceutical industry [44]. The industrial demand for mercury reached its zenith in 1964 and then had a significant decline from 1980 to 1994 due to federal prohibitions on mercury additions in paints and insecticides, as well as a decrease in its application in batteries [45].

### 2.6. Aluminum

Among the elements found in Earth’s crust, aluminum ranks third in abundance [46]. Aluminum is found naturally in the atmosphere, hydrosphere, and lithosphere. The extraction and refinement of aluminum increase its concentration in the environment [47]. Recent studies on environmental toxicity indicate that aluminum poses a considerable risk to humans, animals, and plants, leading to many disorders. The toxicity of aluminum is significantly affected by several parameters, such as water pH and organic matter concentration; when pH decreases, toxicity escalates. The mobilization of hazardous aluminum ions, induced by alterations in soil and water pH due to acid rain and escalating atmospheric acidification, adversely affects the environment. This is evidenced by forest desiccation, phytotoxicity, agricultural decline or failure, mortality of aquatic fauna, and different disruptions in the functioning of human and animal systems [48]. A soil surface layer pH below 5 (pH < 5) can result in soil acidity, a global issue that impacts agricultural yields. Aluminum toxicity limited crop productivity to 67% of the global area of acid soils. Aluminum is among the most abundant elements in Earth’s crust. In acidic soils where solution pH drops below 5.0, silicon is increasingly leached from the soil matrix, facilitating the destabilization of aluminum-bearing minerals and the accumulation of aluminum oxyhydroxides such as gibbsite and boehmite [49]. As pH rises slightly within the acidic range, this octahedral complex hydrolyzes into monomeric species Al(OH)^2+^ and Al(OH)_2_^+^. However, free trivalent remains the primary driver of phytotoxicity. Its extreme toxicity stems from a high affinity for negatively charged cellular targets in the plant root apoplast, plasma membrane, and symplast, causing severe inhibition of root elongation [50]. Plant toxicity from Al^3+^ results from its affinity for binding sites in the apoplast, plasma membrane, and symplast, leading to significant disturbances in both the physical structure and physiological activity. Standard diagnostic signs encompass the suppression of root elongation, cellular deformations in leaves, the production of stunted and dark green leaves, and the onset of yellowing, necrosis, chlorosis, and purpling in foliar tissues [46]. High levels of aluminum are extremely harmful to aquatic life, particularly gill-breathing species such as fish, as they disrupt plasma and hemolymph ion balance, leading to osmoregulatory failure. Fish rely on the gill enzyme to absorb ions. However, this enzyme is rendered inactive by the monomeric form of aluminum [51]. Al toxicity also impacts aquatic life, including crawfish and seaweeds [52]. Aluminum possesses no biological function and is a harmful, nonessential metal to microbes [53]. Enzymes, including hexokinase, phosphodiesterase, alkaline phosphatase, and phospholipase, are inhibited by aluminum due to their higher affinity for DNA and RNA. Aluminum influences metabolic pathways in living organisms that involve the metabolism of calcium, phosphorus, fluorine, and iron. Aluminum is detrimental to neurological, skeletal, and hematopoietic cells [48,54].

### 2.7. Molybdenum

Molybdenum ranks 54th in abundance among the elements found in Earth’s crust, occurring naturally across the lithosphere primarily within molybdenite ores, as well as in aquatic and atmospheric compartments. The expansion of industrial mining, steel alloy production, and chemical pigment processing significantly accelerates its release and concentration in surrounding environmental repositories [50]. Ecotoxicological assessments reveal that while molybdenum functions as an essential micronutrient in trace amounts, elevated concentrations pose a substantial risk to plants, animals and microbes, culminating in distinct physiological disorders [55]. The bio-availability and subsequent toxicity of molybdenum are highly sensitive to environmental parameters, operating inversely to aluminium; when environmental pH increases, molybdenum mobility and toxicity escalate [8]. In alkaline or neutral soils (pH > 6.5), molybdenum converts into highly soluble and bioavailable molybdate anions (MoO_4_^2−^), causing rapid uptake and accumulation in vegetation. This over-accumulation triggers severe phytotoxicity, manifested structurally as agricultural decline, localized chlorosis, golden-yellow discoloration of foliar tissues, and significant inhibition of both root and shoot growth [56]. Leguminous plants tend to uptake more molybdenum, and their contents in seeds are higher compared to other plants [57]. In aquatic ecosystems, high levels of molybdenum disrupt biological homeostasis, compromising survival and inducing metabolic stress in freshwater microinvertebrates, amphibians and fish species [58]. Within biological systems, excess molybdenum possesses an intense affinity for copper binding sites, acting as a potent antagonist that induces secondary copper deficiency, commonly known as molybdenosis or “teart” disease. This condition is particularly detrimental to grazing livestock, resulting in severe neurological impairment, skeletal abnormalities, hematopoietic vascular disruptions, anaemia, and reproductive failure [59]. At the cellular level, excessive molybdenum disrupts essential metabolic pathways by inhibiting critical enzyme groups, including alkaline phosphatase and various esterases, due to structural alterations in their catalytic centers. Furthermore, it alters the systemic balance of sulfur, phosphorus, and iron, leading to profound disruptions in cellular respiration and energy transduction across living organisms. Recent studies have shown that beyond its classic role as an enzyme cofactor, eukaryotic molybdenum homeostasis involves complex transport mechanisms extending past the traditional MOT1 and MOT2 families. Notably, insertional mutagenesis screening in the model microalga *Chlamydomonas reinhardtii* recently identified novel ATP-binding cassette (ABC) family proteins involved in molybdate transport [60]. This discovery establishes the existence of specialized, energy-dependent eukaryotic ABC molybdate transport systems. Elucidating these pathways opens new paths to understand intracellular molybdenum trafficking, partitioning, and homeostatic resilience under trace metal stress in both microalgae and higher plants.

**Figure 2:**
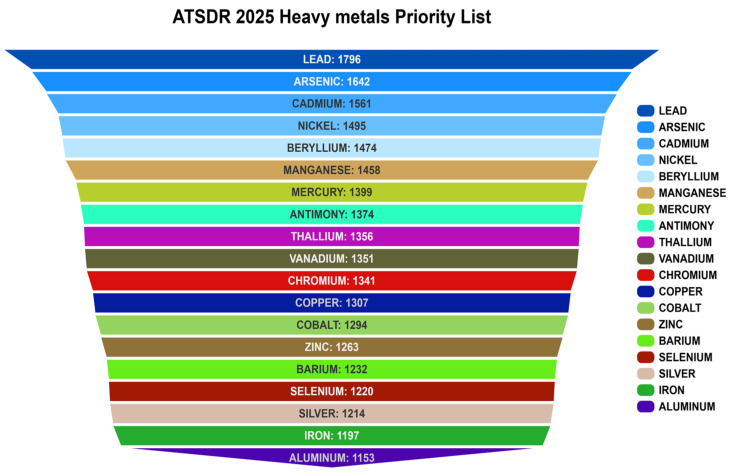
Prioritization hierarchy of major heavy metals in the environment based on public health risk metrics. This categorization reflects the official 2025 Substance Priority List established by the Agency for Toxic Substances and Disease Registry (ATSDR). It should be explicitly noted that this hierarchy does not represent a static ranking of absolute elemental toxicity. Instead, the illustration displays a dynamic environmental risk prioritization determined by a multi-variable algorithm that simultaneously weighs three core criteria including the frequency of substance occurrence at contaminated sites, inherent chemical toxicity, and the overall potential for human food chain exposure. Consequently, elements positioned at the top of the structure represent the most pervasive threats to agricultural ecosystems and human health under standard environmental conditions.

**Figure 3:**
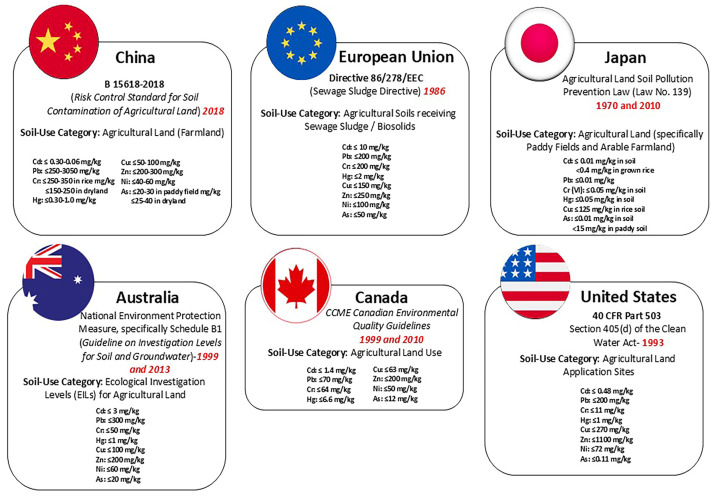
Maximum permissible concentration limits (mg/kg) for heavy metals in agricultural soils across six international jurisdictions. The regulatory baselines and targeted land categories are defined as follows: (1) China: National Standard GB 15618–2018, designating pH-dependent risk screening values for farmland and paddy fields. (2) European Union: Council Directive 86/278/EEC, establishing baseline ranges for agricultural soils receiving sewage sludge. (3) United States: EPA 40 CFR Part 503 Rule, regulating Exceptional Quality biosolid application limits on agricultural sites. (4) Canada: CCME Environmental Quality Guidelines, setting environmental health thresholds for dedicated agricultural land. (5) Australia: National Environment Protection Measure (NEPM 2013 amendment), providing ecological investigation levels for agricultural settings. (6) Japan: Agricultural Land Soil Pollution Prevention Law (updated 2010), targeting arable land and flooded paddy fields to prevent crop dietary contamination.

Figure 2 shows the ranking of heavy metals based on their toxicity as described by the Agency of Toxic Substances and Disease Registry (ATSDR). Figure 3 illustrates that the maximum permissible concentrations of toxic heavy metals diverge significantly across international frameworks due to varying ecotoxicological modelling strategies and localized soil conditions.

## 3. Sources of Heavy Metal Dissemination in the Agricultural Ecosystem

### 3.1. Fertilizers

One of the most prevalent ways heavy metals (loids) are introduced into agricultural soils is through fertilizers rich in macronutrients. However, this is not the case in the poorest developing nations worldwide, where very little fertilizer is used [61]. Nitrogen (N), phosphorus (P), and potassium (K) are known as the “major macronutrients,” the three most commonly used to promote plant growth and production. Nutrient compounds are incorporated into the soil individually, when required, or collectively in diverse combinations known as “compound fertilizers.” Examples of these fertilizers include NPK and NP. The global fertilizer market size in 2020 was estimated at about 171.76 billion USD and 193.28 in 2021 which is predicted to be increased by 2030 up to 241.87 billion USD [62]. Sulfur (S), Calcium (Ca) and magnesium (Mg) are classified as “secondary” macronutrients due to their application as fertilizers; however, calcium is predominantly utilized in the form of lime (commonly CaCO_3_) to elevate the pH of acidic soils, while magnesium may also be employed as dolomite (MgCO_3_·CaCO_3_) for a similar purpose. Alongside these macronutrient fertilizers and liming agents, specific micronutrient fertilizers are applied to the soil or to plant leaves to provide critical trace elements. A diverse array of compounds comprising the elements Mn, B, Ni, Zn, Cu, Fe, Mo and Co is employed for this purpose [63].

The predominant inorganic compounds used in macronutrient fertilizers contain substantial levels of contaminant heavy metals and metalloids; however, this may be beneficial in cases where the “contaminant” serves as a micronutrient, such as zinc, contingent on the quantities provided [64]. Phosphoric acid fertilizers often contain the highest levels of heavy metals, including arsenic, cadmium, uranium, thorium, and zinc. Sedimentary phosphate rock (phosphorite) deposits are recognized for their abundance of various elements, making them the primary source of phosphorus for fertilizers [65]. Due to contaminants in fertilizers, arable soils routinely fertilized may accumulate significant quantities of specific heavy metals, depending on the quality of the fertilizers used. Table 1 shows the ranges of heavy metals across different fertilizers used in agricultural fields.

The most significant disparities in heavy metal (loid) concentrations are observed in cadmium (1700-fold variance), vanadium (800-fold variation), and arsenic (400-fold variation). Phosphorus fertilizers are ubiquitous worldwide, with a distribution factor of 600-fold. The presence of elevated Cd contents in numerous P fertilizers is a considerable concern. In recent years, phosphorus fertilizers made from guano-based phosphorites in Australia have exhibited cadmium concentrations below 300 mg Cd kg^−1^, contributing between 30 and 60 g Cd ha^−1^ to the nation’s soils [80]. Particularly noteworthy is the fact that lime was nearly as high in terms of cadmium inputs to soils in England and Wales as phosphorus fertilizers were. This finding brings up some really intriguing questions. This was not due to high concentrations of cadmium in the lime itself; instead, it is because of higher volumes of lime utilization in soils in comparison to the amounts of phosphorus fertilizers [81]. Elevated zinc concentrations commonly found in phosphorus fertilizers serve as a potent source of this element in sandy and calcareous soils across several regions of the world. Nonetheless, a recent transition to ‘high-analysis’ phosphorus fertilizers, such as monoammonium phosphate (MAP) or diammonium phosphate (DAP), which contain reduced levels of zinc and other heavy metal impurities, will require increased application of zinc fertilizers in the future. Conversely, while the highly refined nature of these fertilizers initially reduced the direct input of heavy metal contaminants into agricultural soils, declaring that they altogether reduce soil pollution is an oversimplification [82]. The high solubility and subsequent nitrification of ammonium-based phosphate inputs can accelerate localized soil acidification. A decline in soil pH often enhances the bioavailability, desorption, and trophic mobility of existing heavy metal pools (such as cadmium or lead) within the soil matrix, while simultaneously increasing the risk of phosphorus runoff into adjacent aquatic ecosystems. Therefore, the environmental impact of high-analysis fertilizers represents a shift in ecotoxicological dynamics rather than a net reduction in pollution, necessitating careful, pH-buffered management strategies [83].

Micronutrient fertilizers that provide important metals, primarily copper or zinc, can significantly increase their levels; however, such fertilizers are often used only when the soil’s available copper or zinc levels are inadequate. In England and Wales, approximately 5% of cereal-producing sites exhibit copper shortages, and the application of copper fertilizer compounds increases the total copper contributed to soils in these nations to almost 28 tons annually [84].

### 3.2. Pesticides

Herbicides target weeds, fungicides target fungi, and insecticides target insects. Pesticides are chemicals or compounds formulated to eliminate or inhibit the growth of pests, including fungi, weeds, and insects. Herbicides target weeds, fungicides target fungi, and insecticides target insects [85]. Historically, numerous acknowledged insecticides widely used in agriculture contained substantial levels of metals. Recently, about ten percent of the compounds authorized for use as pesticides and fungicides in the UK are derived from complexes containing copper, mercury, lead, manganese, or zinc. Examples of copper-containing pesticides include copper oxychloride and Bordeaux mixture, which contains copper sulfate [86]. Among the various pesticides utilized in fruit orchards are phenylmercuric acetate (a fungicide) and lead arsenate (an insecticide). These insecticides include heavy metals, including mercury, arsenic, and lead, which contaminate the environment upon exposure [87]. Arsenic-containing compounds were widely utilized for the management of cattle ticks and insect control in bananas in New Zealand and Australia. Wood has been effectively protected using copper, chromium, and arsenic (CCA) treatments, and numerous neglected sites now exhibit soil concentrations of these metals that are considerably above background levels. The utilization of metal-based pesticides has since been banned due to their toxicity and detrimental effects on human health [86]. Conversely, they substantially contribute to the increase in heavy metal levels in the environment [88,89,90,91]. A significant soil accumulation was recorded by Naccarato at conventional agricultural sites, involving non-essential elements such as Al, As, Ba, Be, Ga, In, Li, Sr, U, and V as well as essential nutrients including Ca, Co, Cu, Fe, Mg, Na, and Zn. This accumulation is indicative of the persistent, long-term use of herbicides and insecticides [92], fungicides [93], and fertilizers [67], which may significantly contribute to the concentration of trace elements in agricultural soils.

### 3.3. Bio-Solids and Manures

The application of various biosolids, including animal manures, composts, and municipal sewage sludge, always results in the accumulation of heavy metals in the soil. Elements classified as heavy metals consist of arsenic (As), cadmium (Cd), chromium (Cr), copper (Cu), lead (Pb), mercury (Hg), nickel (Ni), selenium (Se), molybdenum (Mo), zinc (Zn), thallium (Tl), and antimony (Sb), along with others [94,95]. Manures from chickens, cows, and pigs, generated as byproducts of livestock production, exemplify animal waste frequently applied as solids or slurries on agricultural fields and pastures [96]. While most manures are regarded as beneficial fertilizers, the addition of Cu and Zn as growth boosters in pig and poultry diets, along with the As present in poultry health products, may lead to potential soil contamination with heavy metals. Moreover, the arsenic present in poultry health products may also pose a risk of soil contamination with heavy metals. Animals that ingest such diets produce dung with elevated levels of elements, including As, Cu, Zn, and others [96,97]. Over time, regular applications of this chemical to restricted areas might lead to a considerable buildup of metals in the soil. The term biosolids is increasingly prevalent as an alternative to sewage sludge, as it is seen as a better way to convey the beneficial attributes inherent in sewage sludge. This explains the diminishing use of the term ‘sewage sludge’ [98]. Biosolids, or sewage sludge, are primarily organic solid substances generated as a consequence of wastewater treatment. Crucially, evaluating these long-term accumulation trends exclusively through total concentrations oversimplifies the true environmental threat. The migration of heavy metals into the food chain is governed strictly by their speciation and bioavailable fractions rather than total soil reserves. For example, sequential extraction frameworks demonstrate that biosolid-borne metals are predominantly held in non-exchangeable, stable fractions (e.g., bound to sulfides or organic complexes), restricting initial plant uptake. Nonetheless, natural weathering, root exudates, and the mineralization of the biosolid organic matrix can alter soil solution chemistry, shifting metals into free ionic forms (e.g., Cd^2+^ or Zn^2+^) that are easily absorbed by crops. Thus, future risk assessment models must account for these time-dependent speciation changes [99]. The application of biosolids to land is a common practice in numerous nations that permit the reuse of biosolids generated by urban populations [100]. More than 30% of the sewage sludge collected in the European Community is used as fertilizer for agricultural produce [94]. Lead, nickel, cadmium, chromium, copper, and zinc are the most frequently identified heavy metals in biosolids. Composting biosolids and various organic materials, such as sawdust, straw, or garden waste, has recently attracted considerable interest. This concept possesses significant utility. If this trend continues, there will be repercussions for soil contamination by metals. Metals supplied to soils via biosolids may contaminate groundwater under certain conditions. These metals may be leached downward through the soil profile under specific conditions [101].

### 3.4. Industrial Wastewater and Metal Mining

Wastewater effluents containing heavy metals are most commonly found in areas with recent volcanic eruptions, soil erosion, urban runoff, or airborne particulates. The issue is exacerbated by urban runoff as well. It has come to light that volcanic eruptions can have far-reaching effects on ecosystems, weather patterns, and human health. Not only do eruptions release gases such as carbon dioxide, sulfur dioxide, carbon monoxide, and hydrogen sulfide, but they also release a broad range of organic compounds and heavy metals, including gold, lead, and mercury, which worsen social and chemical conditions. Gases such as carbon monoxide, sulfur dioxide, carbon dioxide, and hydrogen sulfide are part of this category. The obvious consequence of these heavy metals being present in water is a marked decline in water quality. It seems that metals in soils and streams are a result of a combination of rock types and a variety of volatiles that originate from volcanic eruptions. This occurs because water-permeable rocks facilitate the circulation of acidic volcanic gasses, which aids in the hydrological material transfer that occurs in volcanic strata. The formation of volcanic strata is aided by the movement of acidic gasses released during volcanic eruptions. Some sources claim that volcanic eruptions release a wide variety of metals into the atmosphere. Arsenic, mercury, aluminum, rubidium, lead, magnesium, copper, zinc, and a plethora of other metals are all part of this category [102]. Heavy metals end up in wastewater from human activities like electroplating and metal polishing. Mining, extraction operations, the textile industry, and nuclear power are some of the other human activities that contribute to heavy metals. Thin protective coatings are deposited into the prepared surfaces of metal using electrochemical methods as part of electroplating and other “finishing” treatments. Soil metal contamination has been found in many countries as a result of metal resource extraction, processing, and the operations of various industries. Withdrawal involves releasing the larger particles that have collected at the flotation cell’s end into depressions and onsite wetlands, which are already there, leading to a large quantity [103]. The extraction and processing of lead (Pb) and zinc (Zn) minerals have resulted in extensive soil contamination, endangering both human and environmental health. 

Numerous retrieval methods utilized for these sites are laborious and costly, and they may not effectively restore soil efficiency. The ecological hazards that heavy metals in the soil present to humans are linked to their bioavailability. The absorption process may occur via the ingestion of plant matter grown in contaminated soils (via the food chain) or by the direct consumption of polluted soil [104]. Various industries, such as tanning, textiles, and petrochemicals, due to inadvertent oil spills or the use of petroleum-based products, insecticides, and pharmaceuticals, produce extra materials with highly variable configurations. While certain artifacts are discarded through burial, others can enhance agricultural and forestry practices. Moreover, some of them may be detrimental as they contain heavy metals such as cadmium, lead, and zinc or poisonous organic compounds, and they are infrequently if ever, disposed of on land. These chemicals may be detrimental. Conversely, some others possess minimal plant nutrients or exert no influence on soil conditions [96].

## 4. Uptake and Impact of Heavy Metals on Plants 

### 4.1. Uptake of Heavy Metals

Plants known as hyperaccumulators have a remarkable ability to take up heavy metals from the soil, even when those metals are present in varying amounts [105,106,107]. Although hyperaccumulators can absorb heavy metals, the rate of absorption can be influenced by various factors, including pH, water availability, and the presence of organic molecules. Furthermore, the facility must incorporate a suitable transport system for the absorption of heavy metals. Research indicates that pH affects proton secretion by roots, thereby acidifying the rhizosphere and consequently enhancing the dissolution rate of metals and the growth of metal-accumulating plant species [108,109,110]. Organic chemicals emitted from the roots of hyperaccumulating plants can influence development in addition to pH. The organic acids that are generated as a result of Cd complex formation alter Cd’s solubility [111]. Heavy metals are mobilized, and absorption is enhanced by hyperaccumulators’ alterations in pH and the organic compounds released from their rhizosphere [109,111]. A link exists between elevated heavy metal consumption and enhanced root proliferation [112]. The constitutive overexpression of genes facilitates enhanced absorption of heavy metals. Numerous studies have contrasted hyperaccumulating *Arabidopsis halleri* and *Thlaspi caerulescens* with their non-hyperaccumulating congeners to elucidate the genes involved in overexpression. Genes that encode plasma membrane-localized transporters from the ZIP (Zinc-regulated transporter Iron-regulated transporter) family are overexpressed in *T. caerulescens* and *A. halleri*, resulting in increased Zn uptake, as demonstrated by research [113]: ZIP6 and ZIP9 in *Arabidopsis halleri*, and ZTN1 and ZTN2 in *T. caerulescens*. Cadmium absorption was observed to decrease in both genera with an increase in zinc content, offering compelling evidence that zinc governs the expression of ZIP genes [114] and that cadmium influx primarily occurs through zinc transporters, which exhibit a pronounced preference for zinc over cadmium [115]. Increasing evidence suggests that the chemical analog of phosphate, known as arsenate, is delivered into plant cells by the same transporters that are responsible for transporting phosphate [116]. The plasma membranes of root cells of *Pteris vittata* exhibited a higher density of phosphate/arsenate transporters than those of *Pteris tremula* 100, according to the findings of a study that compared the hyperaccumulator *P. vittata* to the non-hyperaccumulator Pteris tremula [117]. This difference may have been caused by constitutive gene overexpression. The plasma membranes of root cells of *P. vittata* demonstrated a higher density of phosphate/arsenate transporters than those of *P. tremula*, according to the findings of a study that compared the As hyperaccumulator *P. vittata* to the non-hyperaccumulator *P. tremula* plants [118].

### 4.2. Toxicity of Heavy Metals to Plants

Some heavy metals, such as mercury, manganese, iron, copper, cobalt, zinc, arsenic, and nickel, do not break down in the environment and instead build up in the soil over time as a result of sewage treatment and industrial waste [119,120]. Despite being critical micronutrients required for the healthy growth and function of plants, a number of heavy metals can simultaneously have adverse effects. They are capable of directly interfering with plant physiology, metabolic activity, growth rates, and senescence [121]. The physicochemical qualities of soil are fundamental to the uptake and accumulation of heavy metals by plants. Heavy metals predominantly accumulate in the root cells of plants due to the potential entrapment by cell walls or obstruction by Casparian strips. The excessive accumulation of heavy metals in plant tissues can directly or indirectly disrupt several morphological, biochemical, and physiological functions, thus affecting agricultural output [122]. Heavy metals diminish crop yield by inducing detrimental effects on several physiological systems in plants, including photosynthesis, seed germination, remobilization of seed reserves, and accumulation during germination and growth [123]. The production of reactive oxygen species (ROS), encompassing radicals such as hydroxyl radicals (OH^•^) and superoxide radicals (O^2•−^), as well as nonradicals such as singlet oxygen and hydrogen peroxide (H_2_O_2_), is one of the most immediate consequences of toxic heavy metals in plants [124,125,126]. To indirectly produce reactive oxygen species, one can use NADPH oxidases (NOXs) to block enzymes via essential cation displacement or to directly produce reactive oxygen species using Haber-Weiss/Fenton reactions with ROS-active metals [123]. In plants, the mitochondria, chloroplasts, and peroxisomes are the primary sites for the formation of reactive oxygen species (ROS). Other sites include the endoplasmic reticulum, the cell wall, and the plasma membrane [127,128].

### 4.3. Effect of Arsenic on Plants

The presence of arsenic (As) in plants is not known to have any biological importance. It is well known that it can cause plants to become poisonous [129]. Arsenic influences the morphological, physiological, biochemical, and metabolic characteristics, including root and shoot length, biomass, chlorophyll concentration, photosynthetic rate, stomatal conductance, transpiration rate, relative water content of leaves, and sugar metabolism, inducing biochemical stress by causing membrane disruption and the production and accumulation of toxic superoxide ions (O^2•−^), hydrogen peroxide (H_2_O_2_), and malondialdehyde (MDA) levels in plant tissues [130,131]. Because arsenic toxicity is more likely to occur in plant cell plasma membranes, it damages plant cells, decreases stomatal conductance, alters nutrient uptake, and disrupts transpiration [132]. Seed priming trials have shown that arsenic toxicity significantly diminished rice seedling germination by 70% and adversely affected growth, decreasing root and shoot length and seedling biomass by factors of 1.3, 1.6, and 1.4, respectively [133]. Extreme arsenic toxicity significantly affects the germination and growth of rice seeds [134]. The major mechanism of arsenic toxicity is its competition with phosphate as its chemical analog in the form of arsenate. Because of the inability of plants to detect these two analogs separately, plants take up arsenate by the same phosphate transporters. Once inside the cell, arsenate competes directly with phosphate in critical metabolic reactions, particularly during oxidative phosphorylation. There are also two other ways in which arsenic harms plants biochemically and molecularly: (1) disruption of cellular redox equilibrium caused by a rise in reactive oxygen species (ROS) formation and accumulation in plant tissues at the same time. (2) the removal of active enzymes or the modification of their ion concentrations [135]. Plants that are subjected to arsenic stress produce reactive oxygen species (ROS) in their tissues. These ROS are characterized by the presence of superoxide anion (O^2•−^), singlet oxygen (^1^O_2_), hydrogen peroxide (H_2_O_2_), and hydroxyl radical (OH^+^). It is dependent on the degree of stress, the metal speciation, and the concentration of the metal in the growth medium, as well as the period, the plant species, and the age of the plant, that the accumulation of ROS causes stress in plants [136].

### 4.4. Effect of Cadmium on Plants

Cadmium is one of the metals that poses a significant threat to living beings, particularly plants. The symptoms of cadmium toxicity are immediately recognizable, and they include chlorosis and stunted growth [137]. Increased toxicity stunts the growth of plants and ultimately results in necrosis [138]. The effects of cadmium toxicity on plants include the inhibition of carbon fixation, the reduction in chlorophyll concentration, and the reduction in photosynthetic activity [139]. Cadmium exposure in soil causes osmotic stress by diminishing leaf-relative water content, stomatal conductance, and transpiration, leading to physiological harm [140]. Cadmium toxicity induces excessive formation of reactive oxygen species (ROS), resulting in damage to plant membranes and the degradation of cellular macromolecules and organelles [141]. The transport and intake of calcium, phosphorus, magnesium, potassium, and manganese are all impeded by cadmium [142]. A number of phytohormones, including abscisic acid, auxin, and gibberellic acid, are responsible for controlling the process of seed germination, which is an essential portion of the plant lifecycle [143]. The presence of toxic quantities of cadmium inhibits plant development and production, inhibits germination, interferes with the physiological processes of seedlings, and decreases agricultural productivity [144,145]. Root necrosis, breakdown, and the development of mucilage are all caused by prolonged exposure to cadmium, which also results in a reduction in root and shoot elongation, as well as leaf rolling and chlorosis [141,146]. Through the disruption of chloroplasts in leaves [139] and the stimulation of ROS growth in the mitochondrial electron transfer chain, cadmium may indirectly contribute to the generation of reactive oxygen species (ROS) [147]. When plants like rice and peas are exposed to Cd, peroxisomes produce ROS through the activation of plasma membrane-bound NADPH oxidase [148]. DNA damage caused by Cd comprises cellular membrane and nucleic acid degradation, damage to photosynthetic proteins, and reduced protein synthesis, all of which affect growth in general [141]. A number of anomalies, including laggards, precocious separation, fragmentation, stickiness, and double and single bridges, are indicators of DNA damage [149].

### 4.5. Effect of Chromium on Plants

Chromium (Cr) is very harmful to plants, disrupting physiological and biochemical processes. Cr exists predominantly as Cr (III) and Cr (VI), with Cr (VI) being more hazardous due to its high solubility [150]. Necrosis, stunted seed germination, stunted root growth, stunted seedling development, reduced biomass production, and chlorosis of the leaves are all symptoms [151]. Numerous plant species, encompassing cereals, legumes, vegetables, forages, and trees, are impacted [152,153,154,155,156]. Chromium decreases seed germination in plants like *Phaseolus vulgaris*, *Cucumis sativus*, *Lactuca sativa*, *Panicum miliaceum*, and *Medicago sativa* [157]. There is a correlation between this inhibition and lower hydrolytic enzyme activity, which in turn restricts the supply of sugar to embryos [158]. Chromium diminishes root and shoot biomass, resulting in leaf abnormalities such as reduced size, chlorosis, and necrosis [159]. Those who are exposed to Cr experience stunted growth, roots that are not fully grown, and necrotic lesions [160]. An excessive buildup of Cr brings about many physiological changes. These changes include a decrease in photosynthetic pigments, a disruption in the balance of nutrients and water, and enhanced carbon absorption and enzyme activity. In plants such as *Ocimum tenuiflorum* and *Phragmites australis*, the presence of Cr(VI) stress influences the production of chlorophyll and carotenoids, which in turn affects photosynthesis [161,162]. There is a similar reduction in the net photosynthetic rate and stomatal conductance that occurs in species such as Gossypium hirsutum and Helianthus annuus when Cr(III) is present [163]. The oxidative stress that is caused by Cr results in the production of reactive oxygen species (ROS), which in turn causes lipid peroxidation and damage to the cells. Sorghum bicolor and Brassica juncea both experience an increase in malondialdehyde (MDA), which is an indication of lipid peroxidation, when they are subjected to Cr(VI) stress [164]. Despite the induction of antioxidant enzymes such as superoxide dismutase and catalase, they frequently prove inadequate in countering elevated chromium levels [165,166]. The drastically higher phytotoxicity of hexavalent chromium Cr(VI) relative to trivalent chromium Cr(III) stems from distinct uptake pathways and intracellular redox behavior. While Cr(III) enters plant roots passively and slowly due to low solubility, Cr(VI) exists as highly soluble chromate CrO_4_^2−^, structurally mimicking essential nutrients to actively hijack endogenous sulfate and phosphate transporters. Once inside the cytoplasm, Cr(VI) acts as a powerful oxidizing agent, undergoing a non-enzymatic reduction cascade back to Cr(III) via highly reactive Cr(V) and Cr(IV) intermediates. This intracellular reduction generates an acute burst of reactive oxygen species (ROS), causing severe lipid peroxidation, enzyme denaturation, and DNA damage [167].

### 4.6. Effect of Lead on Plants

Lead (Pb) is a standard heavy metal pollutant found in soils, and it is found to be particularly detrimental to plant life [168]. Pb may cause a variety of morphological, physiological, and biochemical problems in plants despite the fact that it serves no biological purpose in plants. In most cases, it is absorbed by the roots of the plant, where it accumulates, and only a small portion of it is carried to the aerial parts of the plant [169]. Consequently, root vegetables such as carrots and sweet potatoes may have high levels of lead, followed by leafy greens such as lettuce and Swiss chard, whereas fruits such as tomatoes are less likely to acquire lead from the soil through their roots [170]. Lead accumulation in plants significantly impairs photosynthesis, diminishing the photosynthetic rate, inhibiting chlorophyll synthesis, interrupting the Calvin cycle, and inducing CO_2_ deficit, which results in stomatal closure [171]. Pb(NO_3_)_2_ has a more significant impact on chlorophyll B than it does on chlorophyll A in *Ceratophyllum demersum* because it reduces the photocatalytic activity of chlorophyll and changes the makeup of the lipid compounds [172]. This prevents the growth of flowers and fruits [173]. Lead contamination impairs tomato development, does not influence vitamin C content, and diminishes flower production at elevated contamination levels [174]. A decrease in sunflower plant height, leaf number, and dry matter is observed when lead concentrations are high [175]. However, a rise in the concentration of lead does not result in a decrease in the weight of the fruit in okra [176]. The leaves of blackberries contain 4.5 times more lead than the fruits, and the levels of lead in blackberry leaves frequently surpass WHO standards, providing a significant risk to consumers [177,178].

### 4.7. Effect of Mercury on Plants

In plants, mercury (Hg) toxicity is a serious environmental problem because of the high toxicity of mercury, its ability to stay in the environment, and its widespread pollution. This review focuses on the many different facets of mercury contamination, including its uptake by plants and the harmful effects that ensue from this uptake. Both natural and anthropogenic activities, such as mining, industrial processes, and volcanic eruptions, are the sources of mercury contamination [179,180]. The element mercury can be found in a variety of forms, including metallic mercury (Hg^0^), inorganic mercury (HgS, HgCl_2_), and organic mercury, primarily methyl mercury (MeHg). Of these, the forms that are the most bioavailable and hazardous are mercury(II) and MeHg [181]. Plants predominantly take in Hg through their roots, which are responsible for retaining the majority of the Hg, and only a small amount is transferred to the shoots [182]. Heavy metals can generate significant toxic effects within plants, even at low concentrations. These consequences include the slowing of growth [183], the disruption of photosynthesis [184], the formation of reactive oxygen species (ROS) [185], and damage to lipids, DNA, and proteins [186]. These effects lead to oxidative stress, which plants reduce through diverse defense mechanisms. Enzymatic antioxidants, including peroxidase, catalase, ascorbate peroxidase, superoxide dismutase, and glutathione peroxidase, alleviate Hg-induced stress [187]. In addition, non-enzymatic antioxidants, such as glutathione, phytochelatins, proline, and ascorbic acid, play a role in the detoxification of mercury [188]. 

### 4.8. Effect of Aluminum on Plants

Aluminium (Al) occurs naturally, primarily in aluminum silicates; a minimal quantity of elements is necessary for biological activities in living creatures. Due to its high reactivity, aluminum affects the cell wall, plasma membrane, nucleus, and cytoskeleton of plant root cells [189]. It impacts and interferes with cell walls, plasma membranes, signal transduction pathways, and calcium homeostasis [190]. In tea plants, the epidermal layer cells get thicker as a consequence of aluminum toxicity, which occurs in the older leaves [191]. Exposure to aluminum dramatically elevates proline content in leaves. An observable elevation in malondialdehyde levels, together with stimulation of superoxide dismutase and peroxidase, has been noted in aluminum-treated leaves [192]. Aluminum concentrations in the soil might have an indirect impact on shoots, both with increasing and decreasing levels [46]. Reports indicate that nitrogen, phosphate, and iron contents markedly decline when aluminum concentrations rise in the shoots of maize plants subjected to aluminum stress. Magnesium diminishes markedly at elevated aluminum concentrations (>9 mg/L) [193]. Aluminum toxicity in plants initiates growth inhibition, callose accumulation, cytoskeletal distortion, and disruption of the plasma membrane’s surface charge. Subsequently, aluminum has been demonstrated to impair H+-adenosine triphosphatase (ATPase) activity, induce lipid peroxidation of membranes, and elevate the generation of reactive oxygen species (ROS) in the cytosol and, ultimately, in mitochondria. The damage results in respiratory inefficiency, the opening of mitochondrial permeability transition pores, a collapse of the inner mitochondrial membrane potential, activation of mitochondrial proteases, and the triggering of nuclear apoptosis, culminating in programmed cell death [194].

### 4.9. Effect of Molybdenum on Plants

Molybdenum is required by plants in trace amounts, but it is essential for plant growth, nutrient absorption and yield enhancement. It is a key component of different plant enzymes like nitrate reductase and nitrogenase, which play an important role in nitrogen metabolism and nitrogen fixation in legumes [195]. Molybdenum is also a crucial factor in plant growth due to its involvement in chlorophyll production and stress resistance, especially in crops grown in acidic soils [196,197]. The deficiency of molybdenum could be expressed in different plant symptoms, which can only be treated by the addition of molybdenum, which implies that molybdenum is irreplaceable [198]. The concentration of Mo in plants is significantly correlated with soil properties, including pH, redox potential, organic matter content, and texture. Mo deficiency usually occurs in acidic soils where Mo is less accessible to plants. Occasionally, Mo deficiency can occur in weakly acidic or neutral soils if the original Mo content in the soil is extremely low [199]. Healthy plants maintained a molybdenum level between 0.2 and 2 mg/kg, where it is absorbed in the form of molybdate anions (MoO_4_^2−^). Application of molybdenum disulfide (MoS_2_) in rice increases the chlorophyll content, while at 500 µg mL^−1^ the chlorophyll content index will increase 21.7% in new and 22.4% in old leaves of rice. When plants suffer from a lack of molybdenum, various physiological disruptions occur, resulting in stunted overall growth, dying meristems, poor-quality fruit, and defective flowers. Leaf development is also heavily impacted; foliage may become mottled, leathery, or cupped, with incomplete blade growth leading to the well-known “whiptail” condition in brassicas. Furthermore, this nutrient stress manifests as yellow spot disease in citrus, chlorotic leaves, and premature grain formation in corn [200]. When a plant takes up far more molybdenum than it needs, the toxicity does not always announce itself as a direct chemical burn; instead, it triggers a cascade of symptoms that are essentially a profound copper deficiency in disguise. High concentrations of molybdate in the tissues interfere with copper’s role at the cellular level, effectively locking the plant out of copper even when the soil supply is adequate, a phenomenon well characterized in mineral nutrition research. Inside the cells, the overload generates reactive oxygen species that trigger oxidative stress, damaging membrane lipids and proteins while strongly inhibiting root elongation, which translates above ground into stunted growth and malformed new leaves [201]. 

### 4.10. Toxicity of Micronutrients

**Figure 4:**
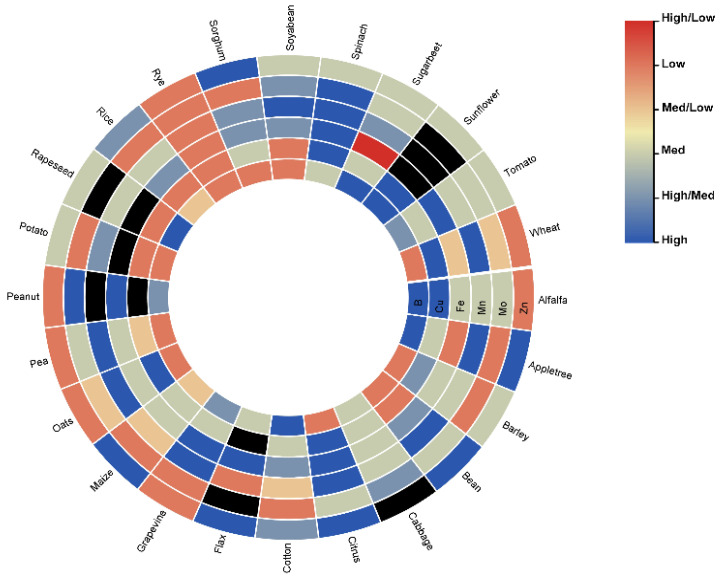
The relative susceptibility of crops to deficiencies of micronutrients. Comparative breakdown of essential trace nutrient functions, deficiency thresholds, and variation in crop susceptibility. Susceptibility levels designated under ‘*Sensitive crops*’ classify species that experience severe yield depressions, irreversible chlorosis, or reproductive failure under suboptimal soil solution concentrations. The black colored areas represent the nonavailability of data regarding crop sensitivity to the specific micronutrient.

Micronutrients are essential nutrients required in minimal yet crucial amounts for the regular and healthy growth of plants and animals. Higher plants necessitate eight micronutrients: Boron (B), Chlorine (Cl), Copper (Cu), Iron (Fe), Manganese (Mn), Molybdenum (Mo), Nickel (Ni), and Zinc (Zn) [202]. Table 2 shows the function, symptoms of deficiency, and adequate levels of these macronutrients in plants. These micronutrients are essential for plant growth, development, and metabolism. Nonetheless, their inadequacies may precipitate several illnesses in plants, therefore diminishing the quality and amount of food. Consequently, studies concerning the role of micronutrients in plants have generated remarkable interest and hold substantial importance for researchers. Crop species exhibit significant variation in their vulnerability to deficits of several micronutrients. Maize, rice, citrus, and fruit trees are especially vulnerable to zinc insufficiency, the most prevalent micronutrient deficiency condition in crops. Small grain cereals, including wheat, barley, and oats, along with citrus and alfalfa, have a pronounced vulnerability to copper deficiency [203]. Nonetheless, while wheat is deemed relatively tolerant to zinc deficit, in numerous regions, particularly those with calcareous soils, zinc deficiency in wheat poses a considerable issue due to its limited availability [204]. The susceptibility of different crops to deficiencies of macronutrients can be seen in Figure 4.

## 5. Remediation Strategies for Heavy Metals in Soils

Soil pollution by heavy metals presents a substantial global risk to human health and the integrity of food production systems. Although certain heavy metal contamination arises from natural geological sources, the predominant portion is attributed to anthropogenic activities, including mining, smelting, military operations, electronic manufacturing, fossil fuel use, inadequate waste management, pesticide application, and irrigation practices. Coal, a prevalent fossil fuel, comprises multiple heavy metals, including mercury (Hg), lead (Pb), cadmium (Cd), chromium (Cr), copper (Cu), cobalt (Co), zinc (Zn), and nickel (Ni), with amounts between 0.1 and 18 mg per kilogram. During coal combustion, these metals are emitted into the environment as vapor, particulate matter in flue gases, fly ash, and bottom ash [211]. Globally, around 5 million locations, encompassing roughly 20 million hectares, are impacted by soil pollution by diverse heavy metals and metalloids [6]. Numerous remediation approaches have been developed over time to manage, eradicate, or repair soils contaminated by heavy metals. These methods encompass surface capping, soil flushing, electrokinetic extraction, solidification, vitrification, and phytoremediation. These techniques are typically classified into five categories: physical, chemical, electrical, and biological remediation, or organized into three primary approaches: containment-based (e.g., capping or encapsulation), transformation-based (e.g., stabilization or immobilization), and transport-based (e.g., extraction or removal). Each technique functions via different mechanisms, presenting certain advantages and drawbacks (Table 3). Moreover, their efficacy and expenses can fluctuate considerably when implemented in practical situations [212]. 

## 6. Future Research Directions and Implications in Agriculture

To systematically visualize and track the heavy metal burdens underlying these systems, Table 1 establishes the literature-based concentration ranges and calculated global averages of priority heavy metal impurities across major agricultural inputs, serving as a critical comparative baseline for remediation targets. By tracking the trajectory of seven priority toxic elements (Cd, As, Al, Cr, Pb, Hg and Mo), we demonstrate that heavy metal stress cannot be mitigated effectively without understanding how these contaminants originate from industrial inputs or how they mimic essential nutrients to hijack root-zone transporter networks (such as IRT1, NRAMP, PHT, and aquaporin NIP channels).

Furthermore, our multi-tiered framework reveals that traditional physical–chemical remediation strategies, while reliable, are increasingly outpaced by targeted, in situ biotechnological innovations. The integration of biochar assistance, microbial engineering, rhizosphere synergies, and engineered nanomaterials represents the frontier of sustainable agriculture. However, moving these technologies from successful lab-scale trials to operational field execution remains the ultimate hurdle in safeguarding global food safety and agricultural resilience.

The prospects in addressing heavy metal contamination in agricultural systems are vast, with numerous research directions that could offer solutions to the growing environmental and health concerns associated with pollution. One promising avenue is the development of more efficient phytoremediation techniques, particularly through the use of hyperaccumulator plants, which have shown potential in absorbing and detoxifying heavy metals from contaminated soils. Further research is needed to explore the genetic and biochemical mechanisms behind these plants’ ability to tolerate high metal concentrations, which could lead to the engineering of more resilient crop varieties. Additionally, the exploration of bioremediation methods, such as using microbial communities to break down or immobilize contaminants, presents another promising field. Researchers are also increasingly focusing on understanding the long-term effects of heavy metals on soil health and biodiversity, with studies that look into how these pollutants affect soil microbiomes and the overall sustainability of ecosystems.

This article provides valuable insight for researchers, policymakers, and agricultural stakeholders by presenting a comprehensive overview of the sources, impacts, and mechanisms of heavy metal accumulation in crops. By highlighting the various ways in which these pollutants enter the food chain, the paper emphasizes the urgency of finding practical solutions. It also informs readers about the ongoing research in phytoremediation and the challenges faced in mitigating heavy metal pollution in soils, encouraging more studies into alternative agricultural practices, sustainable fertilization, and waste management strategies. The detailed examination of specific heavy metals like arsenic, cadmium, and lead in agriculture makes the article a crucial resource for those working to improve food safety, enhance environmental remediation strategies, and ultimately, safeguard both human health and agricultural productivity in the face of industrialization and pollution.

## Figures and Tables

**Table 1:** Occurrence ranges and calculated global averages of priority heavy metal (loid) impurities across major agricultural fertilizers and soil amendments (mg/kg^−1^ dry weight).

Fertilizer Category	Cadmium (Cd)	Lead (Pb)	Arsenic (As)	Chromium (Cr)	Mercury (Hg)	Reference
**MACRONUTRIENT FERTILIZERS**						
Single Superphosphate (SSP)	0.1–150.0 (≈32.5)	1.0–300.0 (≈25.0)	1.0–50.0 (≈11.5)	10.0–500.0 (≈185.0)	0.01–2.10 (≈0.15)	[66]
Triple Superphosphate (TSP)	1.0–120.0 (≈22.1)	2.0–180.0 (≈18.3)	2.0–40.0 (≈9.2)	20.0–450.0 (≈142.0)	0.01–1.50 (≈0.11)	[67,68]
Monoammonium/Diammonium Phosphate (MAP/DAP)	0.1–80.0 (≈14.5)	1.0–50.0 (≈8.2)	1.0–30.0 (≈6.1)	10.0–300.0 (≈95.0)	0.005–0.80 (≈0.06)	[69,70]
Urea	0.01–0.50 (≈0.05)	0.1–5.0 (≈0.6)	0.01–1.20 (≈0.18)	0.1–15.0 (≈2.4)	0.001–0.10 (≈0.01)	[71]
Ammonium Nitrate/Sulfate	0.05–1.20 (≈0.22)	0.2–15.0 (≈2.4)	0.05–2.50 (≈0.52)	0.5–35.0 (≈7.1)	0.002–0.35 (≈0.03)	[72]
Muriate of Potash (MOP/KCl)	0.1–5.50 (≈0.65)	0.5–22.0 (≈4.3)	0.1–8.20 (≈1.2)	1.0–45.0 (≈12.0)	0.002–0.20 (≈0.03)	[69,70,71]
Sulfate of Potash (SOP/K_2_SO_4_)	0.02–2.10 (≈0.31)	0.2–12.0 (≈1.9)	0.05–4.50 (≈0.75)	0.5–25.0 (≈5.8)	0.001–0.08 (≈0.02)	[72]
**SECONDARY & MICRONUTRIENTS**						
Calcium Nitrate	0.01–0.80 (≈0.12)	0.1–8.0 (≈1.1)	0.02–1.50 (≈0.25)	0.2–18.0 (≈3.1)	0.001–0.05 (≈0.01)	[67,73,74]
Magnesium Sulfate/Chelates	0.05–4.00 (≈0.85)	0.5–35.0 (≈6.4)	0.1–12.0 (≈2.3)	1.0–85.0 (≈22.0)	0.005–0.40 (≈0.05)	[67,75]
**ORGANIC AMENDMENTS**						
Livestock Manure	0.1–6.00 (≈1.10)	1.0–60.0 (≈14.5)	0.5–25.0 (≈5.4)	2.0–120.0 (≈35.0)	0.01–0.80 (≈0.09)	[71,75]
Sewage Sludge	0.5–80.0 (≈12.0)	15.0–1200.0 (≈165.0)	2.0–40.0 (≈8.5)	20.0–1500.0 (≈220.0)	0.10–16.0 (≈1.80)	[75]
Compost and Plant-derived Waste	0.05–3.00 (≈0.45)	2.0–150.0 (≈28.0)	0.2–15.0 (≈2.9)	5.0–200.0 (≈42.0)	0.01–0.50 (≈0.04)	[75]
**PESTICIDES**						
Copper Fungi./Bactericides (e.g., Bordeaux Mixture, CuSO_4_)	0.1–5.50 (≈0.82)	2.0–140.0 (≈34.0)	0.5–18.0 (≈3.1)	1.5–65.0 (≈14.2)	-	[76]
Legacy Arsenical Pesticides (e.g., MSMA, Lead Arsenate)	0.02–1.10 (≈0.15)	50.0–2600.0 * (≈850.0)	200.0–8500.0 * (≈2400.0)	0.5–25.0 (≈4.1)	0.02–1.10 (≈0.15)	[77]
Organomercurial Fungicides (Legacy Seed Dressings)	-	0.5–12.0 (≈2.2)	-	-	50.0–1200.0 * (≈450.0)	[78]
Synthetic Insecticides & Herbicides (Formulations using Talc/Clay carriers)	0.01–2.30 (≈0.40)	0.5–45.0 (≈11.3)	0.1–8.50 (≈1.6)	2.0–110.0 (≈26.5)	-	[66,79]

“-” representing the absence of a specific heavy metal within the pesticide formulation under standard scientific screening methods. “*” represents the high extreme values denote formulations where the heavy metal/metalloid acts as the intentional active structural component (ingredient) rather than an unintentional impurity.

**Table 2:** Micronutrients and their functions in plants.

Element	Function	Deficiency Symptoms	Adequate Range	Sensitive Crops
Cobalt (Co)	Crucial for the symbiotic fixation of N_2_ in the root nodules of legumes and certain non-legumes, wherein three enzymes are recognized as co-dependent.	Inadequate nitrogen fixation Chlorosis inhibits growth. Decreased seed production and viability	0.02–0.5 mg/kg	Cereals
Copper (Cu)	Functional and structural roles in oxidative enzymes. These Cu-dependent enzymes and proteins affect photosynthesis, glucose metabolism, respiration, protein metabolism, cell wall lignification (and water transport), and pollen production.	Withering Melanism Twisted white tips Decreased panicle development Disruption of lignification and the formation and viability of pollen	10–25 mg/kg	Foods such as alfalfa, sunflower seeds, spinach, onions, and carrots
Iron (Fe)	Essential for photosynthesis, nitrogen and sulfur utilization, ethylene production, and chlorophyll biosynthesis in plants. It is a key component of cytochromes and catalase enzymes, and in legumes, Fe-containing leghemoglobin regulate oxygen supply to nitrogen-fixing bacteria in root nodules.	Interveinal chlorosis of young leaves	20–1000 mg/kg	Fruit trees (citrus), grapes, peanuts, soya beans, sorghum and calcifuge species
Manganese (Mn)	Manganese (Mn) activates enzymes for oxidation-reduction, affecting lignin, flavonoid, fatty acid, IAA, and nitrogen metabolism. In C4 plants like maize and sugar cane, Mn is needed for CO_2_ assimilation. It is part of two enzymes: the Mn protein in photosystem II for water photolysis in photosynthesis and MnSOD, which protects tissues from oxygen-free radicals.	Spots of chlorophyll The death of immature leaves Decreased viscosity Spots of necrosis on the pea cotyledons	90–200 mg/kg	Cereals, legumes, and fruit-bearing trees (such as apples, cherries, and citrus)
Molybdenum (Mo)	Redox enzymes like nitrate reductase for NO_3_ reduction and aldehyde oxidase for growth hormone production require Mo. It is part of nitrogenase, a bacterial enzyme that fixes N2 in legume root nodules.	Leaf margin chlorosis Leaf “whiptail” Distorted cauliflower curd NO_3_-induced leaf deformation	0.1–5 mg/kg	Brassicae and legumes
Nickel (Ni)	Nickel (Ni) is essential for urease activity and is crucial for plants utilizing urea as their nitrogen source. It supports healthy embryo and seedling vigor in cereals and enhances plant disease resistance. Ni is also a component of hydrogenase, which aids in nitrogen fixation by bacteria.	Leaf tip necrosis (legumes), Chlorosis and patchy necrosis (Graminae)	0.01–5 mg/kg	Pecan, wheat, potato, bean, soya bean
Zinc (Zn)	Zinc (Zn) is crucial for enzymes in carbohydrate and protein synthesis, gene regulation, biomembrane integrity, protection against free radicals, auxin synthesis, and pollen formation. High Zn levels are needed in meristematic tissues and for carbonic anhydrase in photosynthesis, especially in C4 plants. Zn deficiency reduces photosynthesis, causes roots to ‘leak’, and leads to stunting and ‘little leaf’ symptoms due to auxin degradation.	Mostly monocots have interveinal chlorosis. Stunted growth, “Little leaf” Tree rosetting and violet-red leaf tips	10–120 mg/kg	Cereals (especially maize and rice), grasses, flax/linseed and fruit trees (citrus)
Boron (B)	Cell wall formation and stability. Cell division and growth. Membrane function and stability. Sugar transportation. Hormone Regulation and stress response	The stem becomes torn with brown tips and spindle leaves.	10–80 mg/kg	Alfalfa, Apple, Sunflower, Soybean, Cotton, Tomato

Information extracted from [205,206,207,208,209,210].

**Table 3:** Soil remediation techniques and their pros and cons in field application.

Remediation Approaches	Application	Mode of Operation	Advantages	Disadvantages	Status of Application
Surface capping	On-site, high levels of pollution	Physical	Simple to install, economical, and highly secure	Restricted to confined regions and specific geographic locales, degradation of land’s agricultural utility	Commonly practiced
Encapsulation	On-site, high levels of pollution	Physical isolation of the contaminant	Enhanced security, rapid installation	Restricted to a minor, superficial contamination zone, elevated expenses, and degradation of agricultural land utility.	Cleaning up contamination from radioactive nuclides and mixed waste
Electrokinetics	On-site, fine soil, moderate to high levels of pollution	Eliminating pollutants using electricity	Elimination of contaminants with minimum soil disruption	Labour-intensive, inefficient, optimal for fine-textured soils with low permeability.	Pilot demonstrations are in progress.
Soil flushing	On-site, coarse soil, moderate to high levels of pollution	Elimination of contaminants using chemical solutions	Elimination of contaminants, minimal disruption of soil, cost-effective, and easy to install.	Optimal for coarse-textured soils exhibiting high permeability and probable groundwater contamination.	Applications for mixed trash cleanup are few.
Immobilization/ stabilization	On-site, high levels of pollution	Deactivation of contaminants through physicochemical transformation	Cost-effective, simple to execute, prompt outcomes	Metal-specific, transient efficacy, soil contaminants persisting	Temporary remedy that has not been authorized
Phytoremediation	On-site, low to moderate levels of pollution	Removal and/or stabilization of contaminants by vegetation	Widespread public approval, minimal expense, straightforward implementation, and appropriate for extensive contaminated regions	Constrained to superficial contamination within the active root zone, this metal-specific, labour-intensive, and inefficient approach requires multiple growing seasons alongside high-cost handling of secondary hazardous materials.	Pilot demonstrations are in progress.
Bioremediation	On-site, low to moderate levels of pollution	Microbial transformation of contaminants	Economical, easy to execute, and minimal disruption to the soil	Suboptimal efficacy, merely ancillary to primary remediation methods	Not used for the removal of heavy metals
Vitrification	On-site and Off-site high levels of pollution	Thermal vitrification of soil for contaminant deactivation	Highly efficient	Elevated expenses, restricted to a diminutive soil area/volume, treated land, and soil experiencing a decline in environmental functions	Frequently applied
Solidification	On-site and Off-site, high levels of pollution	Deactivation of contaminants through the physical solidification of soil	Rapidly deployable, high-efficiency	Expensive, cultivated land and soil are diminishing ecological functions	Frequently applied
Biochar-assisted remediation	On-site, low to moderate level of pollution	Pyrolyzed organic biomass creates a porous structure with high surface area and functional groups (e.g., carboxyl, hydroxyl), binding metal ions via surface adsorption, ion exchange, and precipitation.	Highly cost-effective and eco-friendly. Sequesters carbon and improves soil structure, water retention, and microbial activity.	Long-term stability of immobilized metals is variable. Aging of biochar over time can release bound metals back into the soil solution.	Fully operational and widely applied in commercial field-scale projects.
Microbial engineering	On-Site and Off-Site, low to moderate contamination.	Genetically modified or selectively cultured bacteria and fungi express specific metal-binding proteins (metallothioneins) or pump systems to biosorb, bioaccumulate, or reduce volatile/highly toxic chemical species.	High specificity for target heavy metals. Minimally disruptive to the natural soil matrix.	Strict biosafety regulations regarding the field release of genetically modified organisms (GMOs). Engineered strains often struggle to survive against indigenous soil microbes.	Primarily at the laboratory and pilot scale; field applications face regulatory hurdles.
Rhizosphere-based approaches	On-Site, low-to moderate contamination.	Utilizes synergistic plant-microbe interactions where root exudates (organic acids/chelators) stimulate native rhizobacteria and mycorrhizal fungi to either immobilize metals in the rhizosphere or accelerate plant root uptake.	Maximizes natural ecological synergies. Sustained, low-maintenance, solar-driven remediation process.	Completely dependent on root-zone depth. Soil chemistry conditions (pH, salinity) strongly limit the survival and performance of rhizosphere microbes.	Operational at the field scale, particularly when combined with agroforestry or non-food crop production.
Nanomaterial-assisted remediation	On-Site, moderate to high-level contamination.	Injection of engineered nanoparticles (e.g., nano-zero valent iron [nZVI], carbon nanotubes) that leverage extreme surface-area-to-volume ratios to rapidly reduce or chemically adsorb heavy metals.	Unmatched, near-instantaneous kinetic reaction speeds. Capable of treating deep subsoils and groundwater plumes in situ.	High manufacturing cost. Potential unknown nanotoxicity risks to native soil biota and long-term soil health due to nanoparticle accumulation.	Emerging field status; transitioning from pilot-scale validation to targeted commercial applications.

The data has been extracted from [10,14,25,104,213,214,215,216,217,218,219].

## Data Availability

No new data were created or analyzed in this study. Data sharing is not applicable to this article.
